# Still too far to walk: Literature review of the determinants of delivery service use

**DOI:** 10.1186/1471-2393-9-34

**Published:** 2009-08-11

**Authors:** Sabine Gabrysch, Oona MR Campbell

**Affiliations:** 1 Department of Epidemiology and Population Health, London School of Hygiene & Tropical Medicine, London, UK

## Abstract

**Background:**

Skilled attendance at childbirth is crucial for decreasing maternal and neonatal mortality, yet many women in low- and middle-income countries deliver outside of health facilities, without skilled help. The main conceptual framework in this field implicitly looks at home births with complications. We expand this to include "preventive" facility delivery for uncomplicated childbirth, and review the kinds of determinants studied in the literature, their hypothesized mechanisms of action and the typical findings, as well as methodological difficulties encountered.

**Methods:**

We searched PubMed and Ovid databases for reviews and ascertained relevant articles from these and other sources. Twenty determinants identified were grouped under four themes: (1) sociocultural factors, (2) perceived benefit/need of skilled attendance, (3) economic accessibility and (4) physical accessibility.

**Results:**

There is ample evidence that higher maternal age, education and household wealth and lower parity increase use, as does urban residence. Facility use in the previous delivery and antenatal care use are also highly predictive of health facility use for the index delivery, though this may be due to confounding by service availability and other factors. Obstetric complications also increase use but are rarely studied. Quality of care is judged to be essential in qualitative studies but is not easily measured in surveys, or without linking facility records with women. Distance to health facilities decreases use, but is also difficult to determine. Challenges in comparing results between studies include differences in methods, context-specificity and the substantial overlap between complex variables.

**Conclusion:**

Studies of the determinants of skilled attendance concentrate on sociocultural and economic accessibility variables and neglect variables of perceived benefit/need and physical accessibility. To draw valid conclusions, it is important to consider as many influential factors as possible in any analysis of delivery service use. The increasing availability of georeferenced data provides the opportunity to link health facility data with large-scale household data, enabling researchers to explore the influences of distance and service quality.

## Background

Every year, more than 500,000 maternal deaths occur worldwide, 4 million newborns die and another 3 million babies are stillborn [1-3]. Nearly all these deaths take place in low- and middle-income countries and most could be prevented with current medical care [1,4].

Most obstetric complications occur around the time of delivery and cannot be predicted. Therefore it is important that all pregnant women have access to a skilled attendant, i.e. someone with midwifery skills, who is able to manage a normal delivery and who can recognize and manage obstetric complications, or refer in time if needed. Skilled attendance at delivery is advocated as the "single most important factor in preventing maternal deaths" [5] and the "proportion of births attended by skilled health personnel" is one of the indicators for Millennium Development Goal 5. Access to skilled delivery care is also crucial to prevent stillbirths and to improve newborn survival [1]. Skilled attendants can perform deliveries either at home, in health centres or in hospitals, but it is argued that the most efficient strategy for lower-income countries is to place them in health centres with referral capacity [6]. In practice, skilled attendance in most countries is synonymous with facility delivery.

A large number of studies on determinants of skilled attendance at delivery have investigated a plethora of potential influential factors. In their review article "Too far to walk" Thaddeus and Maine [7] summarise these factors under their conceptual framework of the three delays. Their focus, however, is on factors "that affect the interval between the onset of an obstetric complication and its outcome" [7], i.e. on care-seeking for obstetric emergencies. Although their third delay can apply to all facility births, there is an implicit assumption in their framework that most births occur at home, which is the norm in settings with the highest mortality, and that the first and second delay occur in response to the need to change the delivery venue because of a complication.

Behavioural theory stresses the importance of defining context for behaviour precisely since the "substantive factors influencing one behaviour are often very different to those influencing another behaviour" and "the most effective interventions will be those directed at changing specific behaviours" [8]. For instance, the determinants of condom use with a regular partner differ from the determinants of condom use with a casual partner [8]. Similarly, we would anticipate that the determinants of preventive care-seeking for delivery (i.e. precautionary seeking of a skilled attendant as women go into labour for anticipated normal delivery) are not necessarily the same as those for emergency care-seeking in reaction to a developing complication.

This paper aims to:

1. Explore the scope of determinants of skilled attendance or health facility use for delivery cited in the literature, including preventive care-seeking for delivery. We concentrate on low- and middle-income countries where facility delivery is not universal.

2. Categorize determinants into a manageable number of themes linked to the conceptual framework, namely: (1) sociocultural factors, (2) perceived benefit or need of skilled attendance, (3) economic accessibility and (4) physical accessibility.

3. Clarify the hypothesized mechanisms of action for each determinant and present likely confounders or effect modifiers.

4. Describe common findings (consistency of the research) and the general direction of the effects if applicable.

## Methods

We searched electronic databases (PubMed and OVID's EMBASE, Global Health, Medline and Health Management Information Consortium) to identify review articles of determinants of delivery-service use (not original articles). We combined search terms related to maternal health care (obstetric delivery, parturition, home childbirth, reproductive health services) with search terms related to service use (health service utilisation, accessibility, medically underserved area, rural health services, choice behaviour) as well as using a term combining both (maternal health services utilisation; detailed search strategy available on request). The two authors screened independently for relevance and only two review articles on determinants of delivery service use in low- or middle-income countries were found: Thaddeus and Maine [7], and Say and Raine [9]. All other hits were either not in fact review articles or focussed on different topics. We used the two systematic review articles to identify individual studies and main themes. Referenced articles as well as articles referencing these reviews were read and checked to ascertain further relevant literature. Both qualitative and quantitative studies investigating determinants of skilled attendance at delivery or of facility delivery were included. No constraints were placed on date or language. The overview of the types of determinants studied, their suggested pathways and the typical findings is comprehensive, however, the studies mentioned are exemplary but not exhaustive of the vast literature published on each determinant. Over 80 studies were read but not all are cited in this article.

The breadth of topic, its context-specificity, the lack of comprehensive index terms and the vast differences in methodology employed, rendered the option of doing our own systematic review of this literature in its entirety impractical. Systematic reviews of observational data are useful when trying to estimate an effect of interest that can be assumed to be independent of context (which is true for most biological effects) or when trying to explore heterogeneity that is thought to be due to a limited range of factors. It is only feasible when looking at a narrow range of clearly defined exposures. Our aim instead was to explore the range of what has been done in the field so far and give an overview of findings, rather than estimating any specific effect or even attempt a meta-analysis. While we could have restricted the review to a limited number of exposures, years or countries, this went contrary to our desire to work out the scope of what has been explored in the literature.

## Results

This review found two previous reviews on the topic: Thaddeus and Maine reviewed the whole range of determinants and established the three delays framework [7], while Say and Raine focussed on the influence of place of residence and socioeconomic status on delivery care [9]. Using these review articles and over 80 original articles, we identified 20 determinants which we grouped under four themes in an adapted framework: (1) socio-cultural factors, (2) perceived benefit/need of skilled attendance, (3) economic accessibility and (4) physical accessibility.

**Figure 1:**
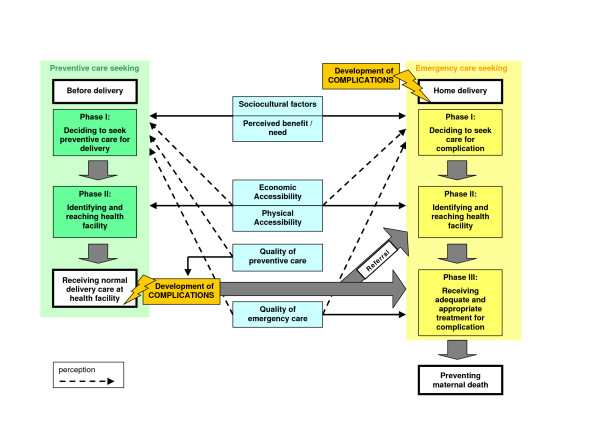
**Delay phases and factors affecting use of delivery care and maternal mortality (adapted from Thaddeus & Maine)**. The three delays for emergency care-seeking are unchanged from the framework presented by Thaddeus and Maine. We conceptually separated preventive care-seeking. Only a first and second phase are relevant for receiving normal preventive delivery care. If a woman who is receiving such preventive care at a health facility then develops a complication, her survival will depend on whether she receives adequate and appropriate treatment in time (third delay of emergency care-seeking). Since she is already in a facility, skilled providers should be able to discover this quickly (no first emergency delay) and she does not need to travel far if it can be handled there (no second emergency delay). For those complications that cannot be handled at that facility and that require referral to a higher-level facility, she will need to travel to a referral facility, possibly with help from the first facility (second emergency delay).

We expanded Thaddeus and Maine's three delays framework to conceptually distinguish emergency care-seeking and preventive care-seeking (Figure 1). While similar factors are involved, their relative importance may differ or they may act in different ways. Cost of transport, for instance, is likely to be a greater deterrent for preventive than for emergency care-seeking. Physical accessibility may exert its role on preventive care-seeking mainly through influencing the decision to seek care, while in the case of emergency care-seeking, reaching the facility in time may be the main problem.

Thaddeus and Maine clearly distinguish between the direct effect of actual accessibility on reaching a facility (second delay) and the indirect effect of perceived accessibility on the decision to seek care (first delay), and correspondingly between actual and perceived quality of care. In Figure 1, we indicate the effects of perceived factors by dashed arrows.

We also changed the categorisation of economic factors in the framework. Thaddeus and Maine group economic status and women's access to money among socioeconomic/cultural factors, a category that is considered to influence decision-making but not the ability to reach a facility. We included economic status in the accessibility category which influences both. The socioeconomic/cultural factors are thus reduced to sociocultural factors, from which we further separated those that influence perceived benefit/need of health facility use. We also split the accessibility category into economic and physical accessibility.

Concerning quality of care, we distinguished quality of emergency care from quality of preventive care. While quality of emergency care – in line with the original framework – is thought to influence the third delay (receiving adequate and appropriate treatment), good quality preventive care for facility deliveries is thought to prevent some complications from arising. Since this literature review investigates determinants of facility use rather than determinants of maternal mortality, the direct effect of quality on the third delay is not relevant. For simplicity we therefore included the indirect effect of perceived quality of care into the category of perceived benefit/need.

While it was important to clarify conceptually how the various influential factors might affect the three delays for both preventive and emergency care-seeking, we did not identify any studies on the topic that distinguished between preventive and emergency care-seeking. However, some studies considered the role of complications in care-seeking and we grouped this determinant with the factors influencing perceived benefit/need.

Table 1 summarises the determinants identified in the literature into the four categories of our framework, with their hypothesized mechanism of action and common findings on their effects. In the following text, we describe the findings in more detail. Where not stated otherwise, we refer to the whole body of literature we reviewed (over 80 articles) when indicating how frequently certain determinants were examined or results found.

**Table 1:** Factors thought to be associated with skilled delivery service use in the literature

Determinant*	**Rationale**	**Findings**
**Sociocultural factors**		

Maternal age +++	Older women: more experienced in using services, more confident, more say in household. Young women: more modern.	No difference, or older women more likely to use services in all multivariate studies examined.

Marital status ++	Single mothers more autonomous: more use. But maybe poorer and stigmatized: less use.	No association or either direction.

Ethnicity, religion, traditional beliefs +++	Certain cultural backgrounds, beliefs, norms and values as well as discrimination may decrease care-seeking.	Mixed results. Large differences in some studies, none in others.

Family composition +	Small children at home and no extended family to help should decrease use.	Some found less skilled care if higher number of births in previous five years.

Mother's education +++	Knowledge, access to written information, modern culture, more confident, higher earnings, control over resources, better communication with husband and providers, etc. should all increase use.	Consistently strong and dose-dependent positive effect on delivery service use.

Husband's education ++	Knowledge, modern attitudes, better communication between spouses, higher autonomy for wife, higher earnings, etc. should increase service use.	Higher husband's education consistently increases skilled attendance; effect often smaller than effect of mother's own education.

Women's autonomy ++	Decision-making power, mobility, control over resources, access to transport should increase use.	Most found some aspects to increase skilled attendance, but others found no effect.

**Perceived need**		

Information availability +	Information about risks of childbirth and about service availability in radio or TV should increase use.	Information access associated with more skilled attendance in some studies but not in others.

Health knowledge +	Knowledge about risks of childbirth and the benefits of skilled care should increase wish to use services.	Expected association in some but not in other studies.

Pregnancy wanted +	Higher value attached to desired child justifies expenses for skilled attendance.	Expected association in some but not in other studies.

Perceived quality of care +	Perceived poor personal and medical quality of care, clash with culture and fear of procedures may decrease use.	Qualitative studies generally find that perceived low quality decreases use, some describe interaction with distance and cost. Very few quantitative studies.

ANC use ++	Familiarity with services, encouragement by health workers increases delivery service use.	Usually those attending ANC much more likely to receive skilled delivery care.

Previous facility delivery ++	Familiarity with services increases their use.	Nearly always very strongly associated with index facility delivery.

Birth order +++	First birth: more difficult, help from natal family, high value on pregnancy, or unplanned/unwanted. High order births: previous experience, confidence if no problems previously, difficulty to leave home with several small children, poorer families.	No difference or first births and lower order births more likely to have skilled attendance than high order births in the vast majority of studies examined.

Complications +	Pregnancy complications (→ ANC advice), complications during delivery, previous complications (→ women aware, medical risk) should increase use of skilled attendance.	Qualitative studies: important factor, decreases importance of other barriers. Few quantitative studies, several found that women with complications are more likely to seek skilled care.

**Economic accessibility**		

Mother's occupation +	Own earnings, range of movement, information should increase use. Decreased use expected if work is poverty-induced.	No effect in several studies, association in either direction. Often less use of skilled attendance among women farmers.

Husband's occupation ++	Higher financial resources and health insurance with some occupations should increase service use.	In several but not in all studies increased skilled attendance if higher status occupations.

Ability to pay +++	Costs for transport, care, opportunity costs decrease use by the poor.	Poorer women less likely to have skilled attendance, in some studies no effect.

**Physical accessibility**		

Region, urban/rural +++	Social and service environment differences between regions. In rural areas generally worse services and infrastructure, more poverty, more traditional beliefs, which all decrease use.	Nearly always moderate to large differentials with less service use in rural areas.

Distance, transport, roads ++	Distance as disincentive and actual obstacle to reach facilities, enhanced by lack of transport and poor roads.	Less service use when further away or no difference.

* Frequency of inclusion in quantitative studies: + rarely, ++ sometimes, +++ usually

### 1. Sociocultural factors

Sociocultural factors primarily influence decision-making on whether to seek care, rather than affecting whether women reach a facility. One could conceptually distinguish the mother's own motivation to use services from whether she can act on her wishes. However, we considered decision-making of both mother and her family and therefore included women's autonomy and husband's education into this category.

#### 1) Maternal age

Age is often presented as a proxy for accumulated experience, including in the use of health services [10-13]. Older women are also possibly more confident and influential in household decision-making than younger women, and than adolescents in particular [10,13,14]. Furthermore, older women may be told by health workers to deliver in a facility since older age is a biological risk factor [10,12,15]. On the other hand, older women may belong to more traditional cohorts and thus be less likely to use modern facilities than young women [13].

Age is highly correlated with parity, and, in some settings, with educational level. It is also associated with marital status, wantedness of a pregnancy, socioeconomic status and decision-making power [16].

Most studies on determinants of delivery service use consider age; those with a multivariate analysis (i.e. controlling for parity) find either no effect of age or a higher use of skilled attendance among older mothers compared to younger mothers.

#### 2) Marital status

Marital status may influence the choice of delivery place, probably via its influence on female autonomy and status or through financial resources. Single or divorced women may be poorer but enjoy greater autonomy than those currently married. Young single mothers may be cared for by their natal family, which may encourage skilled attendance, especially for a first birth. On the other hand, single mothers may be stigmatised and prefer to deliver at home because they anticipate a negative provider interaction [17].

Several studies include marital status and find no association with skilled attendance [18-20], while some find less facility use among married women [21-23]. Studies used a variety of groupings and some did not adjust for confounders, making results difficult to interpret. One study looked separately at monogamously married, polygamously married, never-married and formerly-married mothers in six African countries. Results vary from showing no association (Tanzania, Ghana, Burkina Faso), to monogamous women seeking care more often than the other groups (Ivory Coast and Kenya), to formerly-married and polygamous women seeking more care (Malawi) [24].

#### 3) Ethnicity and religion, traditional beliefs

Ethnicity and religion are often considered as markers of cultural background and are thought to influence beliefs, norms and values in relation to childbirth and service use and women's status. Moreover, certain ethnic or religious groups may be discriminated against by staff, making them less likely to use services [12].

More specifically, women in some cultures may avoid facility delivery due to cultural requirements of seclusion in the household during this time of "pollution" [25] or because of specific requirements around delivery position, warmth, and handling of the placenta. In some cultural groups in Africa, the belief that obstructed labour is due to infidelity hinders care-seeking [7,26]. Beliefs that birth is a test of endurance, and care-seeking a sign of weakness may be another reason for delivering alone in some contexts [27].

In many societies, ethnicity and religion are closely linked to socioeconomic position and place of residence; minority ethnic or religious groups may live in remote areas with worse health infrastructure and transport. [12,18] Inadequate control for socioeconomic position, place of residence or access to services will lead to residual confounding.

Many studies include ethnicity and/or religion, with mixed findings. Most Latin American studies find that indigenous women are less likely to have skilled attendance at delivery [11,12,28-31]. Ethnic minorities in China [32], Kurds in Turkey [33], members of scheduled castes/tribes in India [13], Catholics in Vietnam [34] and non-whites in South Africa [10] are also less likely to receive skilled care. In Ghana, no ethnic differences were detected [18,35], but members of traditional religions and Muslims are less likely to use delivery services as compared to Christians. Several other studies report no ethnic or religious differentials for their settings.

Fewer studies look at beliefs and attitudes directly. Those that do, find that women holding biomedical health beliefs [12], those who had used family planning [36] and those who did not mind being delivered by a male provider [23] are more likely to use skilled providers. Using traditional medicines is not associated with skilled care in two studies [13,37], neither is the presence of ayurvedic providers and traditional birth attendants (TBAs) in the community in a study in Uttar Pradesh [38]. Another study used a high proportion of husbands in the community approving family planning as well as a lower average number of children as measures for modern attitudes and found these to be associated with higher use of facilities for delivery [24].

#### 4) Family composition

Women with young children may have difficulties finding child-care while they deliver at a health facility, in particular if they live in a nuclear family. Sometimes women are accompanied by family members during their hospital stay, so that even these cannot take care of other children during the time [17]. In addition to influencing the ease of leaving home, living with an extended family may also influence decision-making power of the woman; and the number of small children at home may also be a proxy for socioeconomic status, which may be hard to control for.

Few studies consider family composition. Some find a significant influence of the number of births in the previous five years on whether the mother delivered the index birth in a health facility [19,39]. Other studies however do not find any association of preceding birth interval (as a measure of age of the youngest preceding child) [36], of number of children under five in the household [40] or of the ratio children to adults in the household [41] with facility delivery.

#### 5) Mother's education

There are multiple potential pathways that could explain why "maternal education is consistently and strongly associated with all types of health behaviour" [15]. These include increased knowledge of the benefits of preventive health care and awareness of health services, higher receptivity to new health-related information, socialisation to interact with formal services outside the home environment, familiarity with modern medical culture, access to financial resources and health insurance, more control over resources within the household and wiser spending, more egalitarian relationship and better communication with the husband, more decision-making power, increased self-worth and self-confidence, better coping abilities and negotiating skills as well as reduced power differential towards health care providers and thus better communication and ability to demand adequate services [7,10,42-44]. Education also reflects a woman's childhood background, including familiarity with health services and certain beliefs and norms, and some recommend this should be controlled for [11,13,44]. It has also been suggested that there may be community effects of education, with more highly educated communities organising themselves and demanding better public services and a higher position for health on the political agenda [43]. By contrast, better awareness of poor quality in many facilities and higher confidence in self-care may delay care-seeking among educated women. Furthermore where strong public health programs reach out to disadvantaged sectors of the population, the education gradient in health service use may be small.

Education is likely to be associated with wealth and even residence. Adjusting for current wealth will measure the direct effect of education, excluding its indirect effect through improved living standards [45]. It is also important to control for confounding by maternal age since average education levels may have changed substantially over time.

With few exceptions, all studies in the field include maternal education and find a strong and dose-dependent positive effect of educational level on use of skilled attendance, but levels of education are classified differently. For example, in most African settings, effects of primary education versus no education are already well discernable. In Tajikistan, where most women have secondary education and 40% delivered at home in 1998, there is no differential in service use up to secondary education, but those with higher education are more likely to deliver in a facility than the rest [46].

Where the contextual effect of education is considered by including the percentage of women with secondary education in each cluster, it is highly predictive of an individual woman's facility use for childbirth in most of the African countries studied, more so than the also substantial individual education effects [24]. In Haiti and Mali the concentration of adults (not just women) with secondary education is also associated with facility delivery but is restricted to women who had lived in the area for at least 5 years in Mali [40], and in Haiti the association was weakened and lost significance when individual-level variables were added to the model [39].

#### 6) Husband's education

Educated husbands may be more open toward modern medicine [11], aware of the benefits of skilled attendance and more able to communicate with health workers and demand appropriate care, as described for women's education. They may also put fewer constraints on their wives' mobility and decision-making, thus facilitating care-seeking.

Husband's education is associated with occupation and with household wealth. Some studies even use husband's education as their measure of household socioeconomic status [32]. Considerations concerning confounding and pathways are similar to those described for mother's education.

Nearly all studies that consider husband's education find that higher education is associated with skilled attendance at delivery, although the effect is often less than that of the mother's own education.

#### 7) Women's autonomy

The various dimensions of autonomy, such as position in the household, financial independence, mobility and decision-making power regarding one's own healthcare, may all impact on health facility use. In many countries, women cannot decide on their own to seek care, but have to seek permission from a husband or mother-in-law. Furthermore, women may lack control over material resources needed to pay for expenses, their mobility may be restricted or they may lack access to vehicles or even bicycles or donkeys [7,42]. However, women's informal power in the household may mitigate some of the above [7]. The interpretation of various measures of autonomy depends on the context – women who take decisions alone in a context where this is unusual, "might be relatively isolated, unsupported individuals, and not autonomous agents" [42]. As such they may have resource constraints and be less likely to use services.

Women's status, as it reflects on the importance attached to female health also plays a role. "Sex discrimination as a contributory factor to maternal mortality has been largely ignored, [and] has been hidden within the general issue of poverty and underdevelopment which is assumed to put everyone... at an equal disadvantage in health terms." [47]

Autonomy and status effects are likely to be modified by age, marital status, wealth and parity.

Several studies examine the effect of autonomy dimensions on use of skilled attendance at delivery [12,17,20,23,26,36,42,48-51]. Most find significant associations for at least some dimensions, but which ones are important varies from study to study. Dimensions studied include freedom of movement, aspects of decision-making, control over earnings, communication and sharing of housework with the husband, sex of household head and presence of the mother-in-law in the household.

### 2. Perceived benefit/need

This category comprises factors influencing the perception of how a facility delivery with skilled attendance would benefit mother and newborn and/or how big the personal need for such care is. This perception is shaped by general awareness of the dangers of childbirth and interventions available at health facilities, by individual past experiences with pregnancy, childbirth and health services, as well as by risk assessment of the index pregnancy. As for the previous group, factors in this category are thought to primarily affect the decision to seek care.

#### 8) Information availability

Having access to information through modern media could influence women's knowledge about delivery risks and availability of services.

It may be hard to disentangle access to information from possession of radio or TV and the higher socioeconomic status that makes these more likely. Literacy is essential for access to written information.

Several studies examine exposure to radio or TV and to family planning messages in the media [13,24,30,38]. An association with increased use of facilities for delivery is found in some settings but not in others.

#### 9) Health knowledge

Specific knowledge about the risks of childbirth and the benefits of skilled attendance should increase preventive care-seeking, while recognition of danger signs and knowledge about available beneficial interventions should increase care-seeking for complications.

The majority of studies of use of delivery care are cross-sectional and it is difficult to establish time sequence. Contact with a skilled attendant could increase specific knowledge on childbirth via health education. Specific knowledge may also be associated with educational level in general.

Few studies consider health knowledge. Women in Zambia who know danger signs in pregnancy are more likely to deliver in a health facility as compared to those without such knowledge [23] and a similar but not significant tendency was observed in Southern Laos [52]. Also, in Mali, women who are told about complications at antenatal care are more likely to give birth in a facility [40].

#### 10) Pregnancy wanted

Women with unwanted pregnancies may be less likely to invest in skilled attendance at delivery than those who attach high value to the expected child. However, delivery care may be sought due to the risk for the mother rather than the child [44].

Wantedness may be associated with age, parity and social support or marital status.

Wantedness and its impact on uptake of care is rarely studied. A study specifically investigating this question found no association of wantedness with home deliveries in Bolivia or the Philippines, a 20% increase in the odds of home delivery in Peru, a borderline significant increase by 35% in Kenya and a borderline significant decrease by 20% in Egypt [53]. Another study found that wantedness at time of birth increases the odds of having a doctor at delivery by 30% in South Africa while there is no such association in Brazil [10]. In Kenya, the odds of home delivery are increased by 40% when pregnancy is either unwanted or not wanted at that time [36]. No association between wantedness and delivery care was found in Thailand [44].

#### 11) Perceived quality of care

Perceived quality of care, which only partly overlaps with medical quality of care, is thought to be an important influence on health care-seeking. Assessment of quality of services "largely depends on [people's] own experiences with the health system and those of people they know" [7]. Although some elements such as waiting times can be measured objectively, the perception of whether these are a problem and affect quality is more subjective. Elements of satisfaction cover satisfaction with the outcome, the interventions and with the service received – including staff friendliness, availability of supplies and waiting times [7]. In many cases, the medical 'culture' may clash with the woman's, for example, when family members are not allowed to be present, supine birthing position is imposed or privacy not respected; this may lead to perceptions of poor quality [7]. Some studies mention that women report better quality of care in private facilities, but that cost deters them from using those [25,26,54,55].

Perceived interpersonal quality of care overlaps to some extent with traditional beliefs and possibly sometimes with ethnic discrimination. Concerns about quality interact with other barriers, for example with distance or cost. Objective measures of quality of care such as facility infrastructure, equipment and staffing are associated with physical accessibility, access to information and other aspects of remoteness such as poverty and traditional values.

Nearly all qualitative studies of service use in the literature report quality of care to be an important issue, with staff attitudes featuring prominently. Many women report dissatisfaction with rude, arrogant and neglectful behaviour at health facilities and prefer the care of a TBA or relative [26,27,54,56-58]. In several settings women complain about culturally inappropriate care, for example in Hoima district in Uganda providers urge women not to express pain openly [27]. Shortcomings in personal care at facilities are often coupled with shortcomings in hygiene and medical care. Women criticise dirty toilet facilities, lack of water and aseptic practices as well as lack of necessary drugs or too early Caesarean sections [25,27,55,59,60].

Few quantitative studies assess quality of care. A Vietnamese study found that women who delivered in a facility give a significantly higher average quality score for "health care delivery", but not for "communication and conduct of personnel" as compared to women who delivered at home (and who judged these quality aspects from others' experience or earlier contacts with the facility) [17]. Another study in a rural district of Zambia found no effect of perceived quality of care [23] on service use, however, service satisfaction levels were 96%. Facility delivery is associated with higher total number of doctors in the facilities of the area where the woman lives in Uttar Pradesh, but not with staffing levels or drug stock-outs in Paraguay, Uganda or Tanzania [61]. Studies in Morocco and Burkina Faso also found no significant effect of number of health workers or infrastructure on delivery in a facility [62,63]. A survey in Afghanistan also failed to find an effect of presence of obstetric equipment, but equipment levels were shockingly low overall [64].

#### 12) Antenatal care use

Antenatal care (ANC) services can provide opportunities for health workers to promote a specific place of delivery or give women information on the status of their pregnancy, which in turn informs their decisions on where to deliver. Risk assessment during ANC may explicitly recommend a place of delivery, for instance to deliver in a hospital for a twin pregnancy. On the other hand, women who are told their pregnancy is fine may feel encouraged to deliver without a skilled attendant. In Uganda, a study described that nurses abuse women without ANC cards and hinder their admission for delivery services; this deters women who did not use ANC from seeking delivery services [54].

ANC attendance can be a marker of familiarity in interacting with the health system and with the health facility. Women who use ANC may therefore be more likely to use facilities for delivery. Alternatively, use of ANC may signify availability of a nearby service, which may also provide delivery care. In many settings, however, ANC is also provided by mobile clinics and small facilities that do not offer delivery services. Moreover, while timing for ANC is flexible and the service free in most places, this is not true for delivery services.

Any observed association between ANC use and facility use for delivery is always suspect of arising from confounding by other factors, in particular availability of and access to services, since those women closer to facilities are more likely to go to both [24]. Other confounding factors may be knowledge of pregnancy risks and attitude towards health services [15], complications [65] and most other factors influencing service use. When examining the effect of other determinants on use of skilled attendance, controlling for ANC use may be inappropriate as it is likely to be on the causal pathway.

About a quarter of studies investigating determinants for skilled attendance at delivery assess the role of ANC use as a predictor. Some find no effect but most find that women who use ANC are much more likely to receive skilled attendance at delivery. The presence of a health worker providing ANC in the community can also increase use of skilled attendance, as described for Haiti [39]. A study in Mali found that the level of antenatal care uptake in the enumeration area is highly predictive of individual women's health facility use for delivery, even when controlling for individual ANC use [40], which suggests that area-level use may be a proxy for other factors including accessibility.

#### 13) Previous delivery service use

Women who delivered with a skilled attendant previously become more familiar with this setting, which may make them more likely to use it again. Also most determinants, particularly those that do not change (e.g. education, place of residence, beliefs) which influence a previous place of delivery, are likely to operate in the same fashion again. Even more than for ANC, any observed association between previous and subsequent facility delivery use is likely to be confounded by availability of and access to services [24], attitude towards health services [15], previous complications, knowledge about pregnancy risks and various other factors. Naturally, the same determinants that played a role for previous use are likely to influence present use.

Qualitative studies indicate that women tend to deliver with the same provider if a previous delivery went well and tend to change when they are dissatisfied [17,54,56]. Two quantitative multi-country analyses of Demographic and Health Survey (DHS) data found very strong associations between previous and current facility delivery [15,24]. Most odds ratios found by Bell and colleagues are between 20 and 50, while those found by Stephenson et al are not as extreme, probably because the latter controlled for community-level percentage of women who ever had a facility birth as a proxy for service availability and norms [24]. This community level factor is highly associated with place of delivery in five out of six African countries studied [24].

#### 14) Birth order

The first birth is known to be more difficult and the woman has no previous experience of delivery. Often a high value is placed on the first pregnancy and in some settings the woman's natal family helps her get the best care possible [13]. Furthermore, health workers may recommend a facility delivery for primipara. By contrast, women of higher parity, can draw on their maternity experiences and may not feel the need to receive professional care if previous deliveries were uncomplicated [38]. Very high-order births, however, are more risky. Additionally, women with several small children may have greater difficulty in attending facilities due to the need to arrange child care [11,38]. In one setting, referrals for free tubal ligation in public hospitals after delivery were seen as an incentive for older women to seek a facility birth [31], but we interpret this as an effect of higher parity rather than age. In China, the one-child-policy deters women with higher order pregnancies from using services for fear of punishment [32].

High parity may reflect a lack of access to family planning services which may be associated with lack of access to delivery care. High parity can also indicate traditional attitudes, and sometimes lower socioeconomic status which is hard to control for adequately [38].

Most studies in the field consider the effect of parity on delivery service use. The vast majority find higher levels of service use for the first and lower order births as compared to higher order births.

#### 15) Complications

Complications experienced during previous deliveries or loss of the newborn can make women aware of the dangers of childbirth and the benefits of skilled interventions and thus make them use skilled attendance for subsequent deliveries. Furthermore, women with specific medical interventions in a previous delivery, e.g. a Caesarian section, will be encouraged by health workers to seek skilled care for subsequent deliveries since there is an increased risk for rupture with a scarred uterus.

Another possible pathway is that problems experienced during the index pregnancy can make women seek health services antenatally and health workers may then recommend health facility delivery. Finally, complications during an attempted home delivery often influence women and their families to seek professional care, even though the original intention was to deliver at home. Alternatively, a precipitate labour may mean a woman intending to deliver in a facility ends up delivering at home or on the way.

The type and severity of complications that lead to a change in place of delivery depend on the perception of what is abnormal and what is amenable to medical treatment [7]. As mentioned earlier, the factors involved in decision-making are likely to differ for preventive facility deliveries and for emergency care-seeking of attempted home deliveries that run into problems. In the latter case, the severity of complications may override the perception of barriers like distance and cost [7]. Presence of complications could thus be an effect modifier for other barriers. People who consider "normal deliveries" or minor problems as not justifying cost, time and travel to a facility may attempt to overcome those barriers if there is danger to life, even if the cost is much higher [7].

Many studies in settings with low levels of skilled care find that a large proportion of women say they have facility deliveries because they experienced complications [25,59,66]. While few quantitative studies investigate the role of complications, those that do mostly find that at least some types of current or previous complications are associated with health service use for delivery [10,12,31,37,38,48,49,58,62,65,67]. In one study, facility delivery is associated with prolonged labour [37], while another study did not detect any association with prolonged labour or bleeding, but found one with breech delivery [31].

### 3. Economic accessibility

Economic accessibility refers to the relation between financial capability of the family and costs of a facility delivery including transportation costs. While directly affecting whether a woman can actually reach a facility for delivery (second delay), the anticipation of high costs will affect whether a decision for a facility delivery is made in the first place (first delay). We grouped mother's and husband's occupation and other measures of ability to pay in this group, including community-level poverty, although some obviously also measure other aspects.

#### 16) Mother's occupation

Women who are working and earning money may be able to save and decide to spend it on a facility delivery. However, in many settings women either do not earn money for their work or do not control what they earn. An increased range of movement and better access to information are suggested as reasons why formal work may promote women's use of health facilities for childbirth. On the other hand, working may be poverty-induced and indicate resource constraints, which would make working women less likely to use health services for delivery.

Variables associated with occupation may include education, wealth and place of residence and these may act as confounders.

Relatively few studies include women's occupation. Several find that farming women are less likely to have skilled attendance at delivery than women in other occupations [20,35,51]. This may stem from limited financial resources and health services in rural areas – wealth and place of residence were not always adjusted for. A number of studies do not find any effect of maternal working status or occupation [11,17,19,34,68], while others find that formally employed women are more likely to use delivery services [23,30]. In two Southern Indian states [13] and in Nepal [69], however, working women are less likely to use services than non-working women, which may signify that working is poverty-induced in that context. Another study in Bangladesh [70] found an interesting interaction effect: There is a large differential in delivery service use favouring gainfully employed women among those living more than 1 hour travel time from a health centre, while employment status does not play a role among those within 1 hour travel time. This could be due to employed women being better equipped to overcome access barriers including transportation costs or female mobility limitations.

#### 17) Husband's occupation

Wives of husbands with higher status occupations could be more able to use facilities for delivery. High status occupations are associated with greater wealth, making it easier for the family to pay costs associated with skilled delivery care. Certain professions include health insurance benefits, making care-seeking less costly.

Occupation is associated with education and wealth, and these may thus be confounding the relationship. Some studies use husband's occupation as a measure of household economic status [20], but the majority also include other measures such as household assets.

Most studies find that higher status occupation of the husband is associated with skilled attendance at delivery. In rural Haiti, however, a mother is less likely to deliver in a facility when her partner contributes all or part of the household expenses, after controlling for household wealth [39], possibly because she has less autonomy in that situation. A study in Turkey did not find any effect of paternal occupation in itself but when the father had household health insurance, the last birth was more likely to have occurred in a health facility [33].

#### 18) Ability to pay

The cost of care-seeking may include costs of transportation, medications and supplies, official and unofficial provider fees as well as the opportunity costs of travel time and waiting time lost from productive activities [7] (although women in the late stages of labour are unlikely to do any production other than reproduction). Where women do not travel alone, accompanying adults or children for whom no caretaker can be found increase opportunity costs, transportation costs and costs for staying over night in the town where the health facility is located [7]. Households on a tight budget will have great difficulties to pay these costs and therefore be less likely to use a health facility for delivery.

Another reason for greater use of services is that "households with higher living standard are more modern and therefore more receptive towards modern health care services" [13]. On a larger scale, communities with less economic development are likely to be more traditional, give women less autonomy and have less positive attitudes towards service use [38]. An alternative mechanism how economic status affects care-seeking is that the "characteristics of the health facilities serving the poor ... may discourage use" [7]. This may stem from inferior quality of care or worse availability of services in poor areas thus requiring users to travel long distances. "Cost and distance [from a facility] often go hand in hand... as longer distances entail higher transportation costs." [7]

Ability to pay for care-seeking may be associated with modern attitudes and women's autonomy and, on a community scale, with service availability and quality; all these factors are likely to act as confounders.

Nearly all qualitative studies mention cost as an important barrier to formal care. TBAs are usually deemed affordable for poor families since their payment is negotiable in terms of amount and timing and can be in kind [54]. However, Thaddeus and Maine found to their surprise that "the literature indicates that compared to other factors, the financial cost of receiving care is often not a major determinant of the decision to seek care" [7]. On the other hand, they quoted data from Nigeria where a "drastic decline in hospital births" was observed after user fee introduction in the 1980s, while "the admissions of complicated obstetric cases increased" [7]. This suggests that costs deter poorer women from using delivery services for preventive purposes, while they play a lesser role in case of complications where the cost-benefit ratio is different [7]. A study in Afghanistan also found that women living in the catchment area of a fee-charging facility were less likely to deliver with skilled attendance than those living near free facilities, even after controlling for other factors [64].

Nearly all quantitative studies on delivery service use include some measure of household wealth. Most use an asset index; others use single assets such as TV possession or housing material, land size or food sufficiency. While the majority find that richer households are more likely to have skilled delivery care (up to five times more likely), others do not detect an association. This may be partly due to the choice of wealth indicator and of other variables in the model, and partly to household wealth not playing a big role in certain contexts, for example where wealth gradients are shallow, where services are free or where quality is the overriding concern [12,71]. A recent systematic review of the effects of economic status on delivery service use in the literature [9] came to similar conclusions.

A few researchers investigated community-level poverty effects. A study on geographic aspects of poverty and health in Tanzania found that poorer communities (higher percentage of households in the poorest asset tercile) in both rural and urban areas are further away from a hospital, that staffing, equipment and drug supplies in their closest health centre are worse, and that delivery at facilities and with skilled providers is less common [72]. Unfortunately, the authors did not disentangle the effects of infrastructure, community poverty and household poverty. Another study in Haiti found that neighbourhood-level poverty, determined as the percentage of households in the lowest wealth quintile, is associated with decreased use of skilled attendance [39]. In Guatemala, women living in communities with a sewer system, as a measure of community infrastructure, have five times the odds of receiving formal delivery care of those in communities without, controlling for family socioeconomic status, ethnicity, distance to the nearest clinic and various other variables [30]. Similarly, an analysis of urban data in 85 DHS countries found that in most countries, cluster-level living standard strongly influences skilled birth attendance even when controlling for household living standard [73]. Interestingly, this study found that women from poor households living in non-poor clusters have a similar probability of receiving skilled attendance to women from non-poor households living in poor clusters [73], suggesting independent effects of household- and cluster-level poverty.

### 4. Physical accessibility

Like economic accessibility, physical accessibility affects indirectly the first, and directly the second delay. We have included region and place of residence in this category, but realise this is an arbitrary choice since such complex variables also comprise aspects of all the other categories.

#### 19) Region and place of residence

Since "service and social environments are typically very different in urban and rural areas, ... strong urban-rural differences in use of delivery care are expected" [15]. Similar reasoning applies to differences between regions within a country and it can be difficult to know which factor to ascribe any differences in service use to.

Place of residence may be associated with education, ability to pay, parity, ethnicity/religion, beliefs, information availability, autonomy, availability and quality of services and accessibility of services. Its inclusion in an analytic study is therefore questionable if the goal is to disentangle these factors.

The vast majority of studies on delivery service use include region or urban/rural residence among their variables. Virtually all these studies find a large advantage for urban women compared to rural, and even larger for those living in large cities or in the capital. Differentials between regions within a country are usually moderate to large in size. A particularly extreme case is Ethiopia, where the odds of urban women to deliver with skilled attendance are more than 8.5 times, and those of women in Addis Ababa nearly 40 times, those of rural women [19]. A systematic literature review by Say and Raine [9] on the rural-urban difference in delivery service use came to similar conclusions. The only two studies they identified as not showing higher facility use in urban compared to rural women, are one in Kerala [13], where the differential is smaller than in other Indian states (OR 1.7) and not significant, and one that compared urban to peri-urban women in the Kathmandu valley [71]. Mekonnen found evidence for an interaction by place of residence in Ethiopia: While sociodemographic factors influence delivery care in urban Ethiopia, in rural areas distance and travel time are the crucial determinants [74]. Addai suggests a potential interaction of social influence by place of residence: "While all such [individual] choices are bounded by social context, they are probably more so for rural women for whom social, cultural and family ties frame many major decisions" [35].

#### 20) Distance and transport

Distance to health services exerts a dual influence on use, as a disincentive to seeking care in the first place and as an actual obstacle to reaching care after a decision has been made to seek it [7]. Many pregnant women do not even attempt to reach a facility for delivery since walking many kilometres is difficult in labour and impossible if labour starts at night, and transport means are often unavailable. Those trying to reach a far-off facility often fail, and women with serious complications may die en route [7].

The obstacle effect of distance is stronger when combined with lack of transport and poor roads, and its disincentive effect is less pronounced if women have serious complications or the reputation of the provider is good [7]. Even where facilities are conveniently located, they are underused if their quality is considered bad. Where people have the choice between several facilities, they sometimes travel further if the target facility is perceived to offer superior quality care [7,75]. It would thus be useful to consider distance together with service quality and transport options.

It has been argued, that in common with rural place of residence, "distance to hospital also captures other aspects of remoteness such as poor road infrastructure, poor communication between communities, poverty, limited access to information, strong adherence to traditional values and other disadvantages that are difficult to measure quantitatively" [63].

Despite general acknowledgement of its importance, distance or travel time to health facilities is not regularly considered in studies on determinants of skilled attendance, partly due to inadequate data [7,38,39,76]. However, a number of studies have examined the effect of distance. Some also considered road quality, bus services or household transportation means [12,31,39,40].

Many qualitative studies mention distance as an important deterrent from delivering in facilities, in particular when labour starts unexpectedly or at night and in the absence of transport options [17,25,54-56]. A study in Maharashtra [55], however, reported that unexpectedly two women from the remotest village had delivered at a distant private hospital, because "the distance from their village to the primary health centre made them sceptical about delivering at home in the village in case complications occurred" [55].

The vast majority of quantitative studies that include distance report less use of skilled attendance at delivery in women living far away from a facility. Some however find no effect of distance: One such study in Cambodia [37] found that distance from both health centre and hospital had a strong deterrent effect on health facility use for childbirth in bivariate, but not multivariate analyses. The study controlled for birth attendant at the preceding delivery, which is likely to be a very good proxy for physical access to services, potentially better than distance itself which does not contain information on transport options or whether the facilities are functional at all. This may partly explain the loss of significance of the distance variables in the multivariate model. In two other settings where distance does not seem to play a role, the authors reported that health care and transport infrastructure in the area are good [17,58], and thus distance differentials are probably small and unimportant. Even small distances can pose a barrier, however, as shown in Bangladesh [77], when transport difficulties and cultural barriers augment their effect. In the most extreme cases, the odds of having skilled attendance are only one fifth for women in the most distant category as compared to women close-by [37,77].

Two studies reported interesting interactions. Potter found in rural Mexico that road quality ceases to matter when a village is more than 25 km away from a market [31] and Pebley described an interaction with ethnicity in Guatemala [30]: Ladino women living far away from a clinic are less likely to use formal delivery care than those nearby, while there is no such effect for indigenous women. The latter seem to rely on TBAs no matter how close a clinic is, probably due to other barriers. In fact, non-Spanish speaking indigenous women have only 1/100 and Spanish speaking indigenous women 6/100 the odds of ladinas of having formal delivery assistance [30].

## Discussion

This paper provides a revised conceptual framework of the determinants of skilled attendance in low and middle-income countries where care is not universal. It identifies 20 determinants of the use of facility delivery or skilled attendant, grouped in four categories: (1) sociocultural factors, (2) perceived benefit/need of skilled attendance, (3) economic accessibility and (4) physical accessibility. It gives an overview of the factors examined, including the hypothesized mechanisms of action for each determinant and presents likely confounders or effect modifiers. It also summarises some common findings and patterns as well as methodological difficulties and gaps encountered. The paper systematically searched for reviews, and identified and considered 2 review articles and over 80 original articles. However, the breadth of the topic and resource limitations means that some important original articles that did or found something very different may have been missed. We feel that picture overall is comprehensive, but is obviously not 100% complete.

### Summary of findings

Factors most consistently associated with receiving skilled care in multivariate analysis are higher maternal age, low parity, maternal education and higher household economic resources. These sociocultural and economic factors are frequently studied, perhaps because they are relatively easily measured and are included in large surveys such as the DHS, Pan Arab Project for Family Health (PapFam), CDC Reproductive Health Surveys and UNICEF's Multiple Indicators Cluster Surveys (MICS).

Facility use for the previous delivery and ANC use are also nearly always highly predictive of health facility use for the index delivery, however, this may be due to confounding by service availability and other unmeasured factors which influence prior service use. Similarly, the strong differentials in skilled attendance usually observed between rural and urban areas and between different regions are probably due to differences in infrastructure, health care quality, social, economic and cultural factors that are not accounted for.

Complications are an indicator of need for services and as such are associated with high levels of use of facility care and skilled attendants. This applies to current complications, but complications in previous pregnancies may also influence care-seeking in the index pregnancy. Despite the obvious importance of obstetric complications in stimulating care-seeking, its role is rarely investigated, probably "in part because population-based surveys such as the DHS typically do not collect sufficiently detailed information to permit such an investigation" [12]. The existing survey data on reported complications are usually regarded as unreliable in terms of measuring medically diagnosed complications. However, it would be desirable to take obstetric need, even women's perceived need, into account in order to differentiate between women using delivery services for preventive reasons and those seeking emergency care, as influential factors are likely to differ and so will the necessary interventions to improve care-seeking. We hope that our revised framework that conceptually distinguishes these scenarios will be useful in guiding future research.

Women's autonomy and status are also found to play a role in influencing use of delivery care. Their investigation however is hampered by difficulties in measuring the various aspects of autonomy and the context-specificity and likely effect modification by other factors. The impact of marital status is also dependent on the context, and findings show associations in either direction.

Quality of health services is identified as an important determinant of care-seeking by numerous qualitative studies; however it has rarely been included in quantitative analyses. This is partly due to a lack of variation in health care quality in small-scale studies covering few facilities and partly due to a lack of such supply-side facility data in large household surveys like the DHS. Gathering quality of care data from household respondents can lead to "courtesy bias" and bias due to unequal knowledge on quality between women who have given birth in a facility and women who have not. Women cannot be expected to report on the technical quality of care. Therefore, a recent study concluded: "It is recommended that the design of future surveys enables facility-level data on the quality of care to be linked to individual-level data on care-seeking behaviour." [39]

Similarly, a lack of good geographical data linked to household data hampers the investigation of the role of distance and potential interactions of distance with other factors despite wide acknowledgement of the importance to take service availability into account. Where distance data are gathered, mostly through community questionnaires, they are often restricted to the respondents' immediate surroundings and to the nearest facility of any kind – which is not necessarily one that offers delivery services. Nevertheless, the vast majority of studies investigating the role of distance find it to be a strong deterrent of delivery service use.

### Methodological challenges

While some common findings in this literature could be summarised, we do not synthesise the results from the reviewed studies into general conclusions about the relative or absolute importance of the various determinants of skilled attendance use or even attempt a formal meta-analysis. There are three reasons for this.

Firstly, researchers use different study types, sampling techniques and inclusion/exclusion criteria. Skilled attendance is operationalised in different ways and exposure variables are classified differently, which makes the magnitude of effects hard to compare. Secondly, the selection of exposure variables included in the models varies widely and studies use different analysis techniques. Some studies fail to control for important confounders or to adjust for clustering, while others inappropriately include variables on the causal pathway, all of which makes results very hard to compare. It is doubtful whether a systematically applied subjective judgement about the general quality of the studies reviewed would be helpful in making a comparison more informative [78].

The third reason is more fundamental and relates to context-specificity. Even if all methods were identical, it would be naïve to expect the effect of, say, distance in Malawi and Peru to be the same, given that infrastructure, transport options, education level, norms around place of delivery and many other factors differ. In fact, the highly complicated web of relationships and interactions between factors, many of which are hard to measure (e.g. informal payments, staff motivation and community cooperation) makes even exploration of heterogeneity difficult. In particular, the existence of complications may modify the effect of many other determinants but is rarely known. In different settings, the proportion of preventive versus emergency care-seeking will vary and thus the importance of the various determinants.

In order to take context into account when synthesising results, one would need to identify the most important context factors. These could include the average level of care offered in the health facilities accessed (mostly dysfunctional health posts to mostly hospitals offering comprehensive emergency obstetric care), the level of development in the area (influencing infrastructure, in particular transport options) and the presence of a disadvantaged culturally distinct group (e.g. indigenous population in Latin America). This would however be extremely difficult to achieve since most of this information is not easily available from the studies.

Another methodological challenge for any study in the field and thus for this review is that most determinants of care-seeking are not pure concepts, but rather labels on a complex mix of components. Many variables overlap with several concepts and some concepts are hard to measure precisely. This poses difficulties for a multivariable analysis that aims to not just be descriptive but to disentangle which factors are most important. For instance, when adjusting for rural or urban place of residence, this may imply adjusting for accessibility as well as sociocultural and economic factors. Unless these have been well measured and included into the model, it will remain unclear which determinants really are most important.

A further challenge relates to the effect of community-level versus individual-level determinants of care-seeking. There are many ways in which community characteristics can affect the probability of a woman delivering with skilled attendance. These comprise intrinsically group-level attributes such as the urban or rural nature of the community, community attitudes and norms concerning childbirth and characteristics of surrounding health facilities, including accessibility and quality. Furthermore, there are aggregate variables, such as level of poverty or education in the community. Most of these aspects have been mentioned in the respective sections of this review. In the case of aggregate variables, the same determinant can have a different meaning and effect on the community than on the individual level, which has to be considered. Community-level variables are often proxies for a variety of factors, and thus "mixed bag" variables as described above, which means it is difficult to disentangle what the actual determinants are and how they act.

Overall, only a limited number of studies investigating determinants of delivery care include community-level effects at all. As Stephenson and Tsui remark: "Studies on the use of reproductive health-care services have focused on the influence of individual and household characteristics and have largely ignored the influence of community attributes and the characteristics of the health services available." [38] Those that do, use a variety of different and often innovative community-level variables. In Chiapas, for example, intra-community division of political affiliation is highly associated with more home deliveries [28] and in Uttar Pradesh, women in more populous communities are less likely to deliver in a facility [38].

The studies using multilevel models find that delivery service use is highly clustered within families, communities and districts, and that even after adding all covariates to the model, there is still significant unexplained community-level variation [12,24,30,32,36,38,39]. This could be due to measurement error in the included variables or to omission of hard-to measure factors such as health care quality, cost or health beliefs. Correlation within families is larger than within communities and harder to explain by the variables collected, while district-level variation is smallest and best explained [24,30,32]. Magadi and colleagues in Kenya found evidence for complex variation: in communities more than 10 km from a health facility, between-woman variation is larger than in those within 10 km distance, which means individual factors may play a bigger role when accessibility barriers are higher [36].

## Conclusion

In conclusion, studies of the determinants of skilled attendance have concentrated on sociocultural and economic accessibility variables and neglected variables of perceived benefit/need and physical accessibility. It is important to consider as many influential factors as possible in any analysis of delivery service use. From an incomplete picture, invalid conclusions may be drawn. Studies ignoring health service infrastructure have sometimes tended to "blame the victim". For instance, from the strong association between educational level and health facility use for delivery, identified in the absence of data on facility availability, some studies draw the conclusion that promotion of female education and literacy is the most effective measure to reduce maternal mortality [35,79], as if that alone would solve the problem in the absence of adequate and accessible health care in rural areas. Where possible, researchers may want to consider designing studies which measure complications and compare determinants of preventive care-seeking with emergency care-seeking for complications.

The increasing availability of georeferenced data provides a promising opportunity to link detailed health facility data with large-scale household data using a geographic information system (GIS). This could help to explore the influence of geographical distance to delivery services on service use, together with the influence of service quality. So far, only few studies have used GIS technology to determine distance, for example Chowdhury and colleagues in the Matlab surveillance site in Bangladesh [77], but the technology holds great potential.

Furthermore, better conceptual development of quality of care is required as it is not measured well at the moment. For comparison between studies, it would be helpful if studies used a clear analytical plan to test specific hypotheses and collect all necessary confounding variables for that purpose instead of performing data-driven analysis. Moreover, contextual variables should be defined and measured in order to be able to compare results between different settings.

For policy relevance, it is particularly important to investigate those factors that are amenable to change, i.e. health service accessibility and quality. While it is also important to address factors such as women's autonomy and knowledge of danger signs, without accessible health services that can save lives, all other efforts to decrease maternal mortality will be in vain.

## Competing interests

The authors declare that they have no competing interests.

## Authors' contributions

SG and OC searched the databases. SG wrote the first draft of the article and both SG and OC subsequently revised the manuscript and approved the final version.

## Pre-publication history

The pre-publication history for this paper can be accessed here:


